# Interpretation of deep non-linear factorization for autism

**DOI:** 10.3389/fpsyt.2023.1199113

**Published:** 2023-06-22

**Authors:** Boran Chen, Bo Yin, Hengjin Ke

**Affiliations:** ^1^Computer School (Huangshi Key Laboratory of Computational Neuroscience and Brain-Inspired Intelligence), Hubei Polytechnic University, Huangshi, China; ^2^Computer School, Wuhan University, Wuhan, China

**Keywords:** interpretation, autism, fMRI, deep symbolic regression, brain network, factorization

## Abstract

Autism, a neurodevelopmental disorder, presents significant challenges for diagnosis and classification. Despite the widespread use of neural networks in autism classification, the interpretability of their models remains a crucial issue. This study aims to address this concern by investigating the interpretability of neural networks in autism classification using the deep symbolic regression and brain network interpretative methods. Specifically, we analyze publicly available autism fMRI data using our previously developed Deep Factor Learning model on a Hibert Basis tensor (HB-DFL) method and extend the interpretative Deep Symbolic Regression method to identify dynamic features from factor matrices, construct brain networks from generated reference tensors, and facilitate the accurate diagnosis of abnormal brain network activity in autism patients by clinicians. Our experimental results show that our interpretative method effectively enhances the interpretability of neural networks and identifies crucial features for autism classification.

## 1. Introduction

In the rapidly evolving landscape of deep learning, remarkable strides have been made in recent years, evincing exceptional outcomes across an expansive array of applications, such as medical (1–3), vehicular technology (4), image (5), and videos (6). This remarkable feat has been accomplished through the innovative merging of more layers in neural networks, thereby generating complex non-linearities, which have yielded unparalleled results in perception tasks, thereby spurring the interest of researchers across the globe (1, 7). Within the realm of autism spectrum disorder (ASD), the prospect of early detection using deep learning technology presents an immensely auspicious prerequisite for timely intervention and treatment. The far-reaching implications of this breakthrough are staggering and hold the key to revolutionizing the diagnosis and management of autism, potentially unlocking a new era of insights into this complex neurological condition. The ramifications of this cutting-edge technology have the potential to ripple across the entire medical field, yielding transformative applications in other areas of medicine and beyond.

Despite the impressive achievements of deep learning techniques in various applications, the underlying rationales and conclusions remain shrouded in mystery, necessitating the need for greater interpretability from a more detailed and concrete perspective (8). The interpretability of neural networks can provide valuable insights into refining the neural network design, drawing more meaningful conclusions, and deepening trust in the neural network. Within the realm of autism classification, the credibility of the neural network hinges upon its ability to accurately identify the essential characteristics of autism, leading to precise classifications (9). Failure to analyze essential characteristics may lead to decisions based on external factors, noise, or interference, which cannot meet the high standards of medicine due to excessive false positives. Therefore, it is imperative to develop interpretability for neural networks to shed light on the black box of neural networks, thereby enhancing their credibility, reliability, and applicability in various domains (10).

At present, the interpretability of functional magnetic resonance imaging (fMRI) data primarily relies on the elucidation of features and classification models. In this regard, the literature has generated noteworthy contributions in both domains, which are summarized as follows. For example, in the early stage of model interpretability upon fMRI data, interpretation of weight vectors of linear models in multivariate neuroimaging was proposed to determine the origin of cognitive functions and associated neural processes (11). From then on, the saliency features activated by the classifier were utilized to interpret reliable biomarkers associated with identifying ASD (12). Subsequently, interpretability could aid in detecting patterns in fMRI data that indicate the presence of autism (13), leading to more accurate diagnoses and personalized treatment plans. Recently, the integrated Gradients (IG) and Deep LIFT techniques were utilized to identify the correlations between brain regions that contribute most to the classification task (14). Nowadays, a hybrid deep learning framework was proposed to improve classification accuracy and interpretability simultaneously (15). By unlocking the power of interpretability in neural networks, we can harness the full potential of these technologies and ensure that they are not only effective but also trustworthy and ethically sound.

However, there exists a lack of research when it comes to ascertaining the interpretability of deep neural networks for fMRI data in autism. The present state of research is marked by a qualitative interpretation, single attention, and limited scope, particularly as it pertains to static aspects. However, it must be acknowledged that there is a palpable absence of interpretability of model dynamics in theory. The intrinsic dynamics of data and the interpretability of models are intimately linked. Comprehending the inherent laws and structure of data can aid in the creation of more precise and dependable analytical models. Concurrently, to truly grasp the driving mechanism behind the data, the model itself must attain a certain level of interpretability. As such, characterizing the intrinsic dynamics of data is a vital objective in data analysis and modeling. To accomplish this goal, various methods have been proposed, including deep learning, symbolic regression, and deep symbolic regression. Deep learning, a technique based on artificial neural networks, can learn the intrinsic structure and patterns of data through multiple layers of non-linear transformations (16) and has achieved remarkable success in fields such as image recognition, speech recognition, and natural language processing. Symbolic regression, a technique based on evolutionary algorithms, can learn interpretable mathematical expressions from data by searching the possible function space to find the function expression that best fits the data (17). Deep symbolic regression amalgamates the strengths of deep learning and symbolic regression to learn interpretable mathematical expressions from data and unveil the dynamic laws behind it. By integrating deep learning and symbolic regression, it can effectively circumvent overfitting and underfitting issues while enhancing the interpretability of the model (18).

To this end, as a result of our antecedent endeavors, we have managed to successfully extract the intrinsic non-linear factors inherent within fMRI via fusing the deep learning and tensor factorization, namely Deep Factor Learning model on a Hibert Basis tensor (HB-DFL). Moreover, our team has endeavored to construct a classifier that can attain the paramount goal of accomplishing accurate discrimination of Attention Deficit Hyperactivity Disorder (ADHD) (19) and Parkinson's (20). This study will extend our previous works on quantifying the correlation between attention features and models, elucidating the dynamic interpretation of models to ensure precise discrimination of autism.

## 2. Methodology

**Figure 1:**
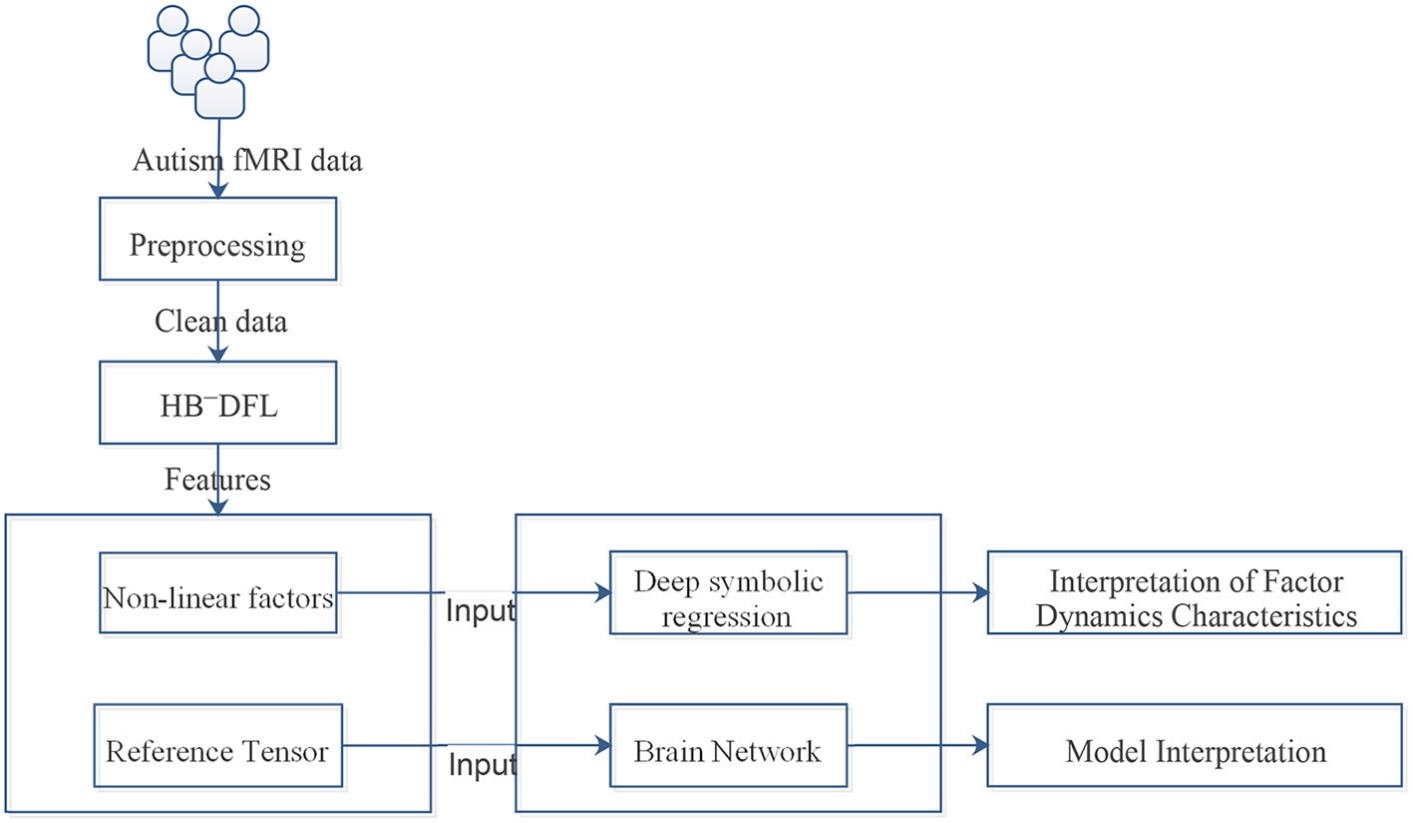
The workflow of the whole solution.

The workflow of the entire solution is illustrated in Figure 1. It is a complex and convoluted process that involves multiple steps of preprocessing, including skull stripping and correction, to ensure the accuracy of the results. The fMRI frames are then sliced and fed into HB-DFL (20), a multi-branch convolutional neural network, where they are mapped to a low-rank space in various directions, generating non-linear factors. To generate a reference tensor equivalent to the initial tensor being decomposed, a pre-defined “constant” kernel tensor is used to perform tensor product operations with the non-linear factors. The difference between the reference tensor and the initial tensor being decomposed serves as the driving force for HB-DFL backpropagation. HB-DFL is then trained using the mean square error loss function until the backpropagation algorithm converges. The desired non-linear factors are then outputted, providing a powerful tool for analyzing fMRI data (19, 20).

This section aims to shed light on the intricate dynamics and relationships between the extracted factors involved in Section 2.1, while providing a comprehensive interpretation of the model in terms of the brain network (Section 2.2).

### 2.1. Deep symbolic regression

**Figure 2:**
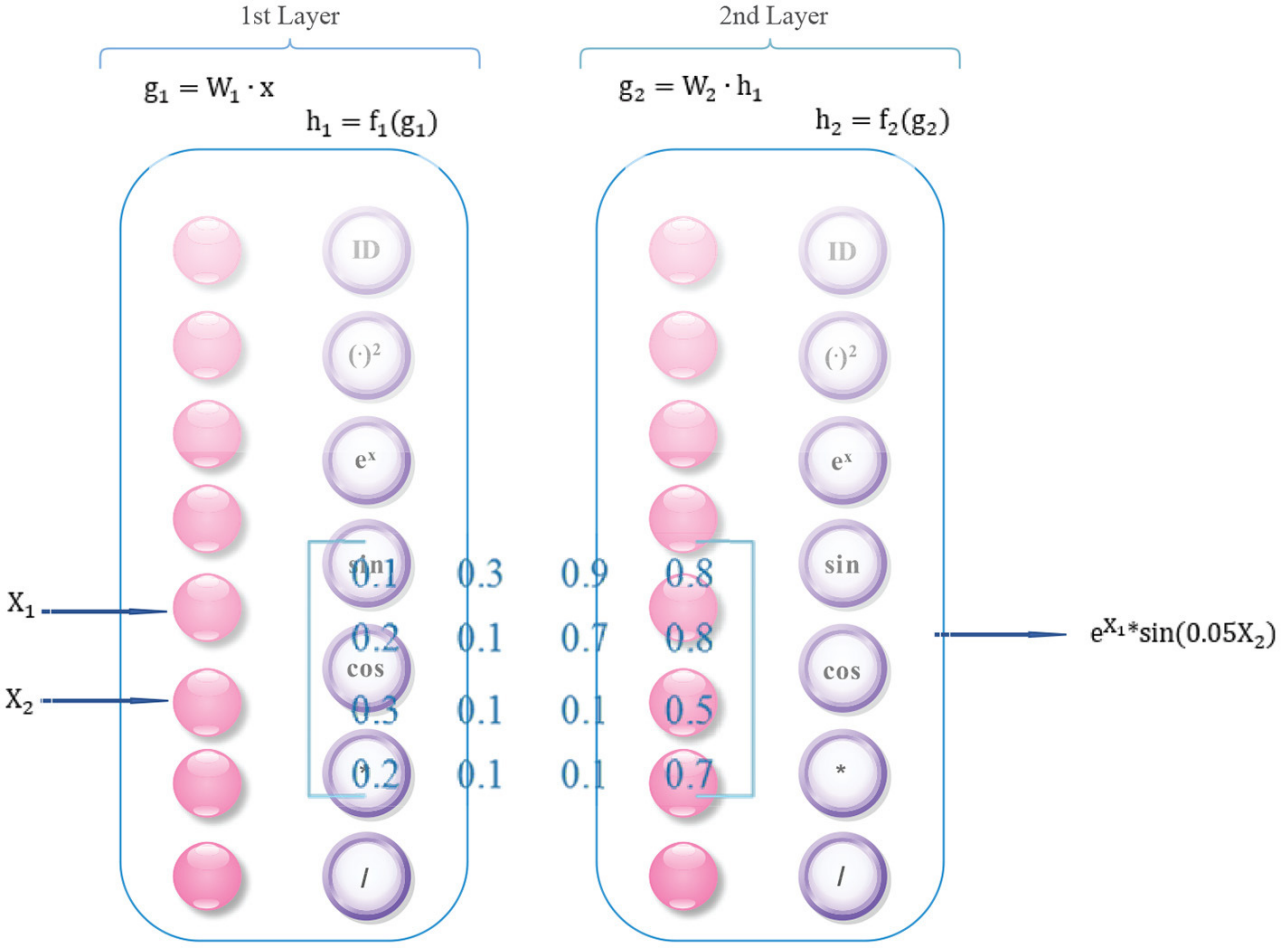
Deep symbolic regression network architecture (two layers as an example).

To better understand the intricate inner workings of complex data, a approach has been extended from deep symbolic regression (18). It involves combining deep learning with symbolic regression techniques, which can yield several benefits. This method capitalizes on the advanced high-dimensional and non-linear processing abilities of deep learning while simultaneously leveraging the compactness, interpretability, and generalization properties of symbolic models. Ultimately, this integrated approach enables efficient pattern discovery in high-dimensional spaces in an end-to-end manner. We achieve the extension of deep symbolic regression by adding more operators, such as differential operators and divide operator. The architecture is illustrated in Figure 2.

Firstly, the factor matrix learned by HB-DFL must be reorganize into a triplet format consisting of (i, j, value) tuples. The row and column indices, represented by i and j, respectively, specify the position of each element within the matrix, while the value component holds the actual value of the element. Once the matrix has been transformed into this format, it can be input into a deep symbolic regression network.

In addition, each layer of the network contains not only regular neurons (g = W • x) but also operators *f*: ID, +, -, *, /, (•)^2^, *e*^*x*^, *sin*(•), *cos*(•), relu, and differential operators: *y*′ etc.


(1) $$\begin{array}{l} f\left(\text{g}\right)=\left[\begin{array}{c} f_{1}\left(g_{1}\right)\\ f_{2}\left(g_{2}\right)\\ \vdots \\ f_{n_{h}}\left(g_{n_{g}-1},g_{n_{g}}\right) \end{array}\right] \end{array}$$

Then, design the optimization function (18):


(2) $$\begin{array}{l} L=|y_{i}-{ŷ}_{i}{|}_{F}^{2}+\lambda L_{\frac{1}{2}}^{*} \end{array}$$

where $|•{|}_{F}^{2}$ denotes the Frobenius Norm. The *y*_*i*_ and ŷ_*i*_ represent the ground truth and predicted label, respectively. $L_{\frac{1}{2}}^{*}$ is the regularization term:


(3) $$L_{\frac{1}{2}}^{*}(w)=\{\begin{array}{ll} |w{|}^{1/2} & |w|\geq a\\ {\left|-\frac{w^{4}}{8a^{3}}+\frac{3w^{2}}{4a}+\frac{3a}{8}\right|}^{1/2} & |w|<a \end{array}$$

where *a* is a predefined constant threshold with the same setting following the strategies of (18) (*a* = 0.01).

Deep Symbolic Regression networks share the same training technique with other deep neural networks, relying on the familiar backpropagation algorithm. Despite this, these networks exhibit a distinguishing trait they build a regression framework that correlates elements of the factor feature matrix with their corresponding subscripts. In other words, given any input row coordinate *i* and column coordinate *j*, the network's output forecasts the element's value ŷ_*i*_, and the residual error between the predicted and actual value *y*_*i*_ determines the update of network parameters.

Finally, based on the combination and arrangement of operators, as well as a predetermined coefficient threshold (≥0.01), the algorithm converges and outputs a mathematical formula in a forward direction according to the network structure.

### 2.2. Brain network

The present study employs a multi-step approach to investigate brain connectivity in disease states. Firstly, temporal information is captured by splitting the tensor into 3D fMRI frames, and factor matrices are obtained using the HB-DFL method previously developed by the authors (20). Subsequently, fMRI frames are reconstructed through a tensor product of factor matrices and Hilbert basis tensors, which are then merged to form a 4D reference tensor. The regions of interest (ROIs) are defined using the automated anatomical labeling (AAL) method, and subsequently parsed into eight distinct subnetworks based on underlying anatomical structures, namely the Default mode network (DMN), Auditory network (AN), Visual network (VN), Soma movement network (SMN), Bilateral edge network (BiN), Subcortical network (SCN), Cognitive control network (CCN), and Cerebellum network. To quantify the connectivity between all ROIs, an entropy-based partitioning approach is employed (21), whereby the mutual information between two brain regions R1 and R2 is derived from the entropy H(R1) and H(R2), and joint entropy H(R1, R2). Based on a threshold value, a connectivity matrix of size 116 × 116 is constructed, and ANCOVA analysis is used to compare the connectivity matrices of control and disease groups at a significance level of *p*-value of 0.05 and false discovery rate (FDR) correction. The proposed approach has demonstrated the potential to provide a more comprehensive understanding of brain connectivity in disease states.

## 3. Results

The experiments in this section serve as a validation and assessment of the interpretability of the proposed model. We first introduce the dataset and experimental platform utilized in the experiments (see Section 3.1). Next, the dynamics of the factor matrix learned by HB-DFL are analyzed through deep symbolic regression (see Section 3.2). Finally, the effectiveness of HB-DFL is evaluated by analyzing the functional connectivity of fMRI data reconstructed using the reference tensor generated by HB-DFL (see Section 3.3). In the following section, we will provide a detailed description of the autistic fMRI data required for these experiments. The experimental procedures were conducted on a single desktop system comprising an Intel i7 CPU clocked at 3.33GHz, an Nvidia RTX 2080Ti GPU, and 64GB RAM running on a 64-bit Windows 7 operating system. This system served as the primary computational platform for all experimental activities, ensuring consistent and standardized testing conditions.

### 3.1. Dataset

The current investigation validates and assesses the proposed approach by utilizing the ASD fMRI dataset publicly available from the Kennedy Krieger Institute (KKI) site of the ABIDE II dataset.^1^ DPABI (22) software is utilized to preprocess both the original fMRI data and the fMRI data produced by HB-DFL. To be more specific, for all fMRI data, fMRIPrep generates brain voxels following skull stripping while disregarding magnetic field inhomogeneity correction. Later on, bbregister (FreeSurfer) is employed to align T1 images to T1W reference images. Before applying spatiotemporal filters, FSL 5.0.9 is utilized to evaluate head motion parameters, and ICA-AROMA is applied to remove motion or noise components. Finally, the images are resampled into two spaces, including the fsaverage5 space and the standard MNI152NLin2009cAsym space.

### 3.2. Dynamic interpretation of factor matrices

This study employs HB-DFL to perform multidimensional tensor factorization on fMRI frames, aiming to investigate the underlying mechanisms of the brain. The resulting factor matrices are randomized and divided into training (80%) and testing (20%) sets. The Deep Symbolic Regression method is used to learn the formula of the factor matrices in the training set, and the performance is evaluated based on the testing set. Table 1 presents the mathematical formulas and verification accuracy of different factor matrices learned from various groups. The study's findings show that most of the mathematical formulas derived from the data are straightforward and have an efficacy rate exceeding 90%, providing evidence of the effectiveness of the proposed method. Overall, this approach using HB-DFL allows for the dynamic interpretation of autism fMRI data with multidimensional attributes and enables the investigation of the underlying mechanisms of the brain, contributing to the advancement of research in this area. The results presented in this study underscore the essentiality of this approach for extracting meaningful information from fMRI data.

**Table 1:** Mathematical formulas and verification accuracy of different factor matrices learned from different groups (Autism group and Normal control group) in KKI fMRI datasets, where y′ represents the first-order differential.

**Mode**	**Group**	**Equation**	**Accuracy (%)**
Coronal plane	Autism	$0.14cos\left(sin\left(log|-0.165cos\left(\frac{X_{1}}{0.05}\right)|\right)-0.11X_{2}\right)$	91.3
			
	Normal	$\frac{1}{\sqrt{\left|\left(\frac{relu\left(sin\left(0.624-\frac{1}{X_{2}}\right)\right)}{0.568}\right)+X_{1}-5.29\right|}}$	90.61
Sagittal plane	Autism	$cos\left(\sqrt{\left|27.709+relu\left(sin\left(X_{1}+\frac{1}{cos\left(\sqrt{\left|4.71X_{2}\right|}\right)}\right)\right)\right|}\right)$	93.41
		Normal	$cos\left(e^{0.017log\left(\left|X_{1}X_{2}-4.2X_{2}\right|\right)}\right)$	92.15
Axis plane	Autism	0.431y'-0.055 cos($\frac{1}{X_{1}e^{cos\frac{1}{X_{1}}}}+X_{2}$)	94.25
		Normal	$sin\left(cos\left(17.65\sqrt{\left|log|X_{1}|\right|}\right)sin\left(X_{2}\right)\right)$	91.74

The original tensor $\underline{X}=\underline{G}\times_{1}f(A)\times_{2}f(S)\times_{3}f(C)$, which is based on the HB-DFL multidimensional tensor factorization theory (20), can be understood as a composite function comprising factor matrices from each dimension and the core tensor $\underline{G}$. By obtaining the dynamics of the factor matrices *f*(**A**), *f*(**S**), and *f*(**C**) when $\underline{G}$ is known, the composite function can be utilized for the dynamic interpretation of Autism fMRI data with multidimensional attributes. The direct extraction of mathematical formulas from fMRI frames yields a maximum verification accuracy of only 70% with highly complex formulas, highlighting the essentiality of factor decomposition via the HB-DFL utilized in this study.

### 3.3. Brain network of reference tensor

**Figure 3:**
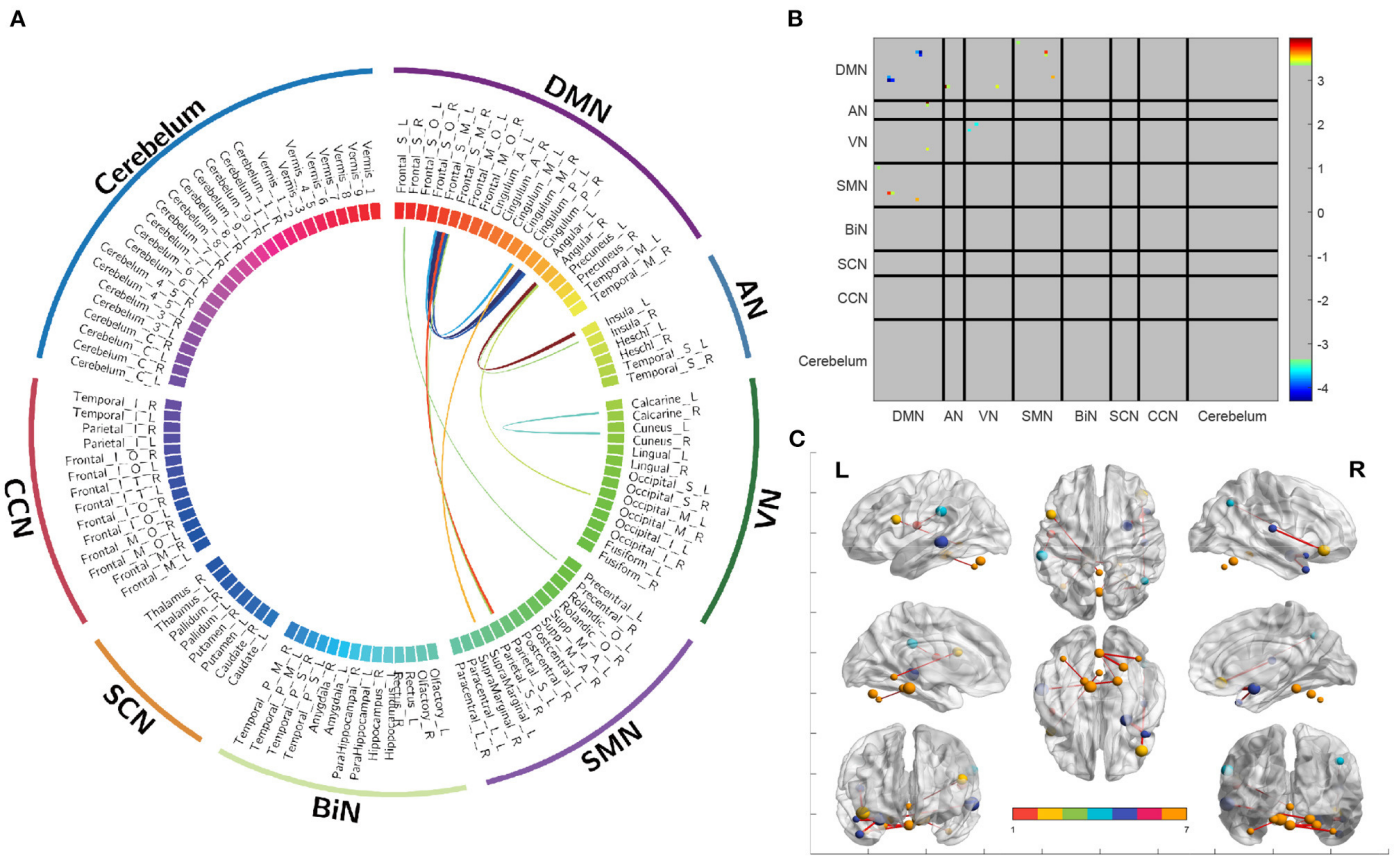
Differences in functional connectivity between different brain regions and their associated subnetworks. **(A)** A circular graph depicting differences in functional connectivity. **(B)** A connectivity matrix showing the partitioning of subnetworks. **(C)** A functional connectivity map of the cerebral cortex.

The utilization of HB-DFL methodology in this study allowed for the analysis of brain functional connectivity networks using a substantial number of reference tensors reconstructed from the KKI dataset. The resulting brain network demonstrated significant functional connectivity and was further analyzed. Figure 3A presents the functional connectivity of subnetworks and the differential connectivity of corresponding brain regions, which revealed noteworthy findings. Additionally, connectivity matrices of subnetworks (Figure 3B) and cortical brain regions (Figure 3C) provided insight into significant statistical differences in functional connectivity between different groups of regions of interest (ROIs). These results provide valuable information on the functional connectivity of the brain, and contribute to the advancement of research in this area.

The findings of the present study replicate those of three prior investigations. Insufficient activation was observed in the DMN (23) and VN (24) of individuals with ASD, in terms of functional connectivity within the network. Enhanced functional connectivity was observed in individuals with ASD between the DMN and VN (25), DMN and AN (26), and DMN and SMN (27), in terms of functional connectivity between networks. Additionally, enhanced functionality was observed between the angular gyrus and insula (28) at the functional brain region level. These results illustrate the effectiveness of HB-DFL in compressive sensing, thereby maximizing information extraction.

## 4. Discussions and conclusions

Autism, a neurodevelopmental disorder characterized by social interaction and communication difficulties, as well as repetitive and stereotyped behaviors, is often studied using fMRI to investigate brain activity patterns. However, the complexity of fMRI data presents significant challenges, such as high dimensionality, noise, and redundancy, which make data interpretation and analysis difficult. To address these challenges, this paper proposes a model based on HB-DFL and deep symbolic regression techniques, which reduces the dimensionality of fMRI data and explains its dynamics. By representing high-dimensional data as a combination of low-dimensional latent factors, the HB-DFL can provide interpretable and understandable factors, each corresponding to a specific pattern of activity in a group of neurons, which can help us understand changes in brain activity in individuals with autism.

The factor matrix, the core component of the HB-DFL, contains the low-dimensional representation of brain activity for each participant and can be interpreted as specific brain networks or functional patterns. The mathematical formula of the factor matrix can thus help us identify and explain changes and abnormalities in brain activity in individuals with autism. However, as a case study, the validation of a single dataset is insufficient to demonstrate the generalizability of the method. To address this, the proposed method will be applied to a wider range of datasets, and cross-validation between different datasets will be performed in the future to demonstrate its robustness.

In conclusion, the proposed model based on deep non-linear decomposition provides a valuable approach to understanding patterns of brain activity in individuals with autism. The interpretability of the model through the mathematical formula of the factor matrix can help us better understand the pathophysiology of autism, and may contribute to the development of future treatment and intervention strategies.

## Data availability statement

The original contributions presented in the study are included in the article/supplementary material, further inquiries can be directed to the corresponding author.

## Author contributions

HK contributed to the conception of the study, the reagents, materials, and analysis tools, conceived and designed the experiments, and analyzed the data. BY and BC performed the experiments. All authors contributed to the article and approved the submitted version.

## Footnotes

^1^ http://fcon_1000.projects.nitrc.org/indi/abide/abide_II.html
